# Serum Adropin Level in the Early Period of ST-Elevation Myocardial Infarction and Its Relationship With Cobalamin and Folic Acid

**DOI:** 10.7759/cureus.32748

**Published:** 2022-12-20

**Authors:** Mehmet Şahin Adıyaman, Revşa Evin Canpolat Erkan, İlyas Kaya, Özlem Aba Adıyaman

**Affiliations:** 1 Department of Cardiology, Gazi Yaşargil Training and Research Hospital, Diyarbakır, TUR; 2 Department of Biochemistry, Dicle University Faculty of Medicine, Diyarbakır, TUR; 3 Department of Internal Medicine, Gazi Yaşargil Training and Research Hospital, Diyarbakır, TUR

**Keywords:** stemi, cobalamin, folic acid, biomarker, adropin

## Abstract

Background: Studies on biomarkers in the diagnosis of myocardial infarction are ongoing. Adropin is a biomarker that has been studied and has been shown to have different effects. This study aimed to examine the adropin level of patients with myocardial infarction within the first 24 hours, as well as its relationship with cobalamin and folic acid.

Material and methods: The control group included 70 patients whose troponin values did not increase and no coronary lesions were detected. In the ST-elevation myocardial infarction (STEMI) group, 70 patients with ST elevation on ECG and coronary total thrombosis on coronary angiography were evaluated. Coronary lesion severity was measured using the SYNergy between the percutaneous coronary intervention (PCI) with TAXUS and Cardiac Surgery (SYNTAX) score tool. Hemogram, troponin, adropin, C-reactive protein (CRP), cobalamin, folic acid, and other biochemical parameters were evaluated in all patients.

Results: In the STEMI group, a significant increase was observed in the adropin level along with the troponin and CRP levels in the first 24 hours (p<0.001). Cobalamin and folic acid levels were low in the same group (p:0.016, p<0.001). While a strong negative correlation was observed between adropin and cobalamin, no correlation was found with other parameters.

Conclusion: The study supports that adropin could be used as a cardiac biomarker in the early stages of STEMI patients. Another result is with low cobalamin and folic acid levels in patients with myocardial infarction which needs to be further explained with the strong negative correlation between adropin and cobalamin.

## Introduction

Acute coronary syndrome (ACS), a clinical condition caused by myocardial ischemia due to a sudden deterioration in the blood flow of a coronary artery, is still an important cause of mortality today. Acute ST-elevation myocardial infarction (STEMI) is a component of ACS. The initiating event of STEMI is often the cracking or rupture of a coronary atherosclerotic plaque. Exposure of the subendothelial matrix with this rupture leads to platelet activation, thrombin formation, platelet aggregation, and fibrin development [1]. STEMI is detected using an electrocardiogram (ECG) and cardiac biomarkers such as troponin [2]. Recently, several studies have been conducted on new cardiac biomarkers. One of the biomarkers studied for this purpose is adropin [3]. Adropin is a 4.9 kDa peptide encoded by the Energy Homeostasis-Related Gene (*Enho*) located on chromosome 9 in humans [4]. Although it is found in various organs in the central nervous system, it is expressed in the heart, kidney, liver, pancreas, and human umbilical cord [5]. It has been observed that adropin has a protective role in the regulation of atherogenesis and cardiovascular diseases, and serum adropin levels are low in patients with stable coronary artery disease [3,6]. Studies have also shown that adropin has regulatory effects on glucose and lipid homeostasis, insulin resistance, impaired glucose tolerance, diabetes, obesity, and hyperhomocysteinemia [4,6]. Adropin levels have been found to vary in cardiovascular studies.

In the current study, we aimed to evaluate the serum adropin levels in the first 24 hours in STEMI patients to enlighten possible pathophysiological mechanisms and investigate its relationship with cobalamin and folic acid. In studies, serum adropin levels were significantly associated with hyperhomocysteinemia in patients with coronary artery disease. It was thought that this relationship might be related to cobalamin and folic acid. The data collected would be used to determine whether adropin is a risk factor for myocardial infarction and its utility as a cardiac biomarker in early diagnosis.

## Materials and methods

Our study was planned as a prospective, single-center, and observational study. Ethical committee approval was obtained for the study. The Clinical Research Ethics Committee at Gazi Yaşargil Training and Research Hospital issued approval number 374. A total of 140 patients were evaluated within the scope of the study. The patients included were given information about the study and an informed consent form was signed. Newly diagnosed ACS patients who presented to the emergency department with chest pain and had ECG changes were included in the study. Patients with a previous history of hypertension, hyperlipidemia, diabetes mellitus, chronic kidney disease, liver failure, and obesity were excluded. The patients were divided into two groups, the control group and the STEMI group. Seventy patients with non-specific ECG changes, no increase in follow-up troponin values, and normal coronary anatomy in the first 24 hours of coronary angiography were included in the control group. In the STEMI group, 70 patients who were diagnosed with STEMI with ≥2 mm of ST-segment elevation in any of the adjacent leads on the ECG and who underwent primary percutaneous coronary intervention due to a thrombus causing 100% narrowing in one or more coronary arteries in immediate coronary angiography were included. SYNergy between the percutaneous coronary intervention (PCI) with TAXUS and cardiac surgery (SYNTAX) scores were calculated in the coronary angiography [7]. All patients included in the study had a blood workup within the first 24 hours. Demographic information and a hemogram were obtained. For blood biochemical analysis, the blood was centrifuged in a flat tube and the blood serum was separated. Adropin, C-reactive protein (CRP), troponin, cobalamin, folic acid, lipid profile, and other routine parameters were evaluated in blood serum biochemical analysis. For the adropin test, the blood was drawn into biochemistry tubes then centrifuged and the serum was separated and stored in a deep freezer at -40ºC. The enzyme-linked immunosorbent assay (Human adropin ELISA) method was used for the adropin test. Serum adropin levels were analyzed in accordance with the kit content obtained commercially, and measurements were made in the Grifols Triturus automated ELISA analyzer device (Grifols, Barcelona, Spain).

Statistical analysis

Statistical analyses were performed using SPSS statistical package program, version 22.0 (IBM Corp., Armonk, NY). Numerical data obtained in the study were expressed as median (minimum-maximum), and categorical data were expressed as frequency (percent). Compliance of numerical data with normal distribution was tested with the Shapiro-Wilks test and homogeneity was tested with the Levene test. Mann-Whitney U and Chi-square tests were used according to the type of data and distribution. Spearman correlation analysis was applied to the data in the STEMI group, which did not show normal distribution. p<0.05 value was accepted as statistical significance.

## Results

Parameters examined within the scope of the study in the control and STEMI groups are given in Table 1 together with their p values. A total of 59 female patients, 43 (72.9%) in the control group and 16 (27.1%) in the STEMI group, were evaluated in the study. Similarly, a total of 81 male patients, 27 (33.3%) in the control group and 54 (66.7%) in the STEMI group, were evaluated in the study (Table 2). It was observed that males were more predominated in the patient group. Adropin levels, together with troponin and CRP levels, were significantly higher in the STEMI group than in the control group (p<0.001). It was observed that the SYNTAX score was higher in the STEMI group compared to the control group without coronary lesions. Low-density lipoprotein (LDL) cholesterol level was high in the STEMI group, while high-density lipoprotein (HDL) cholesterol level was low.

**Table 1:** Parameters studied in the control and STEMI groups and the respective p values CRP: C-reactive protein, HDL: high-density lipoprotein, LDH: Lactate dehydrogenase, LDL: low-density lipoprotein, SYNTAX: SYNergy between PCI with TAXUS and Cardiac Surgery,  Wbc: White Blood Cell, VLDL: very-low-density lipoprotein

Parameters	Control Group (n:70)	STEMI Group (n:70)	p Value
Adropin (pg/ml)	40,00( 40,00-440,00)	203,00 (102,00-640,00)	<0.001
Troponin (ng/mL)	0,10 (0,07-0,13)	12,45 (1,00-25,00)	<0.001
Cobalamin (ng/L)	315,50 (151-728)	291,50 (111-576)	0.016
Folic Acid (ng/mL)	7,30 (3,00-16,20)	5,70 (1,50-20,00)	<0.001
CRP (mg/L)	2,00 (1,00-4,00)	4,15 (0,40-34,50)	<0.001
Age (years)	44,50 (37-68)	54,00 (32-83)	<0.001
SYNTAX score		20,50 (8,00-35,50)	
Wbc count (10^3/uL)	7,33 (4,69-11,60)	13,80 (6,20-19,68)	<0.001
Neutrophil count(10^3/uL)	4,20 (2,24-8,70)	10,31 (4,17-21,60)	<0.001
Lymphocyte count(10^3/uL)	2,16 (1,20-3,55)	2,21 (0,69-5,95)	0.98
Hemoglobin (g/dl)	14,10 (11,00-24,30)	15,04 (11,00-18,10)	0.03
Hematocrit (%)	43,35 (36,00-53,10)	45,50 (36,40-54,90)	0.98
Platelet count (10^3/uL)	265 (156-401)	254 (111-398)	0.17
LDH (U/L)	191,50 (106-280)	351,00 (145-4012)	<0.001
Triglycerides (mg/dl)	84,50 (34,00-179,00)	96,50 (37,00-293,00)	0.067
LDL cholesterol (mg/dl)	93,50 (46,00-130,00)	117 (48,00-183,00)	<0.001
HDL cholesterol (mg/dl)	42,75 (26,20-62,70)	39,25 (22,30-63,30)	0.023
VLDL cholesterol (mg/dl)	20,50 (12,00-40)	25,00 (11-50)	0.001

**Table 2:** Gender assessment between groups

			Group		p Value
			control	STEMI	
Gender	female	count	43	16	<0.001
		% within group	61.4%	22.9%	
	male	count	27	54	<0.001
		% within group	38.6%	77.1%	
Total	count		70	70	
	% within group		100.0%	100.0%	

In the STEMI group correlation analysis exhibited a negative significant correlation between the adropin level and the cobalamin levels (*r *Spearman: -0.337, p=0.004). There was no significant correlation between the other parameters of the group. Information concerning the correlation analysis performed in the STEMI group is given in Table 3.

**Table 3:** Spearman correlation analysis of Adropin with other parameters in the STEMI group CRP: C-reactive protein, HDL: high-density lipoprotein, LDH: Lactate dehydrogenase, LDL: low-density lipoprotein, SYNTAX: SYNergy between PCI with TAXUS and Cardiac Surgery,  Wbc: White Blood Cell, VLDL: very-low-density lipoprotein

Parameters	n	r_Spearman_	p Value
Troponin	70	0.093	0.44
CRP	70	0.146	0.22
Age	70	0.185	0.12
WBC	70	0.033	0.78
Hemoglobin	70	-0.067	0.58
Hematocrit	70	-0.073	0.54
Platelet	70	0.204	0,09
Cobalamin	70	-0.337	0.004
Folic Acid	70	0.092	0.45
LDL cholesterol	70	0.226	0.05
HDL cholesterol	70	0.115	0.34
Triglycerides	70	0.046	0.70
VLDL cholesterol	70	0.062	0.60
SYNTAX score	70	-0.047	0.70

## Discussion

The immunoreactivity of adropin has been detected in many tissues, including the heart [8]. It is suggested that it is an important endocrine factor that affects metabolic regulation, insulin sensitivity, and endothelial functions [4,9]. It plays a role in the maintenance of endothelial vascular homeostasis by increasing nitric oxide synthesis through the endothelial nitric oxide synthase enzyme that it upregulates. Due to this studies suggest that it may have protective effects on the pathophysiology of cardiovascular diseases [5,10]. In the study of Lian et al., in which they compared heart failure patients and healthy individuals, it was determined that the level of adropin increased in proportion to the severity of heart failure, while the level of adropin was low in the healthy control group [11]. They suggested that high serum adropin level plays a role in the pathogenesis of heart failure. In an animal study conducted by Aydın et al. on rats, it was observed that serum adropin levels increased one hour after an experimentally induced myocardial infarction (MI) [12]. On the other hand, in the study conducted by Yu et al., it was found that the serum adropin level decreased in patients with acute myocardial infarction [13].

These differently reported results support the data we obtained in our study. In our control group, who had chest pain but no coronary problems detected by coronary angiography, adropin levels were found to be lower compared to the STEMI group with coronary lesions. Serum adropin levels were found to be elevated in the first 24 hours of STEMI patients with coronary thrombosis and a high SYNTAX score. Considering the studies reporting the protective feature of high adropin levels from cardiovascular diseases, the high adropin levels we detected in STEMI patients may be suggestive of a protective increase. As a result of its association with increased levels of troponin, CRP, and other inflammatory parameters, it has been suggested that it can be used as a biomarker in the early diagnosis of acute myocardial infarction. The results also support studies reporting that it has inflammatory properties. However, the inability to establish a significant correlation in any direction in the correlation analysis suggests that it may not be as effective as the specified parameters in determining the prognosis of the disease.

Various information has been presented in studies on the effects of adropin on the pathophysiology of cardiovascular diseases. In the study by Zhao et al., it was suggested that a low serum adropin level is significantly associated with hyperhomocysteinemia in patients with coronary artery disease [6]. During the synthesis of methionine from homocysteine by remethylation, 5-methyltetrahydrofolate acts as a methyl group donor, while the methionine synthetase enzyme involved in the reaction uses cobalamin as a cofactor. Because of their roles in the remethylation pathway, cobalamin and folic acid may explain the relationship between adropin and hyperhomocysteinemia. The low cobalamin and folic acid levels combined with the high adropin levels we detected in STEMI patients emerged as data supporting this notion. In addition, a significant negative correlation was observed between adropin and cobalamin in the correlation analysis. Although our results showed a strong association between adropin and cobalamin and folic acid in coronary artery disease, the cause of this needs to be further explored. It seems possible to clarify this relationship with more comprehensive studies to be conducted.

Limitations of the study

There were differences in terms of age and gender in the control and patient groups formed in our study. However, in some of the previous studies, no significant relationship was found between serum adropin level and age and gender [14]. It is possible to obtain more reliable information with larger study groups and follow-up adropin values in patients. During MI, follow-up adropin values were not measured in the patients.

## Conclusions

The present study supports that adropin could be used as a cardiac biomarker in the early stages of STEMI patients. However, the inability to establish a significant correlation could show that it does not have a strong relationship, especially in determining prognosis. Another result obtained is the low cobalamin and folic acid levels in patients with myocardial infarction and the strong negative correlation between adropin and cobalamin, which needs further enlightenment.
